# Mapping the 3D position of battery cathode particles in Bragg coherent diffractive imaging

**DOI:** 10.1107/S1600577523000814

**Published:** 2023-02-16

**Authors:** A. G. Shabalin, M. Zhang, W. Yao, R. Rysov, Z. Ren, D. Lapkin, Y.-Y. Kim, D. Assalauova, N. Mukharamova, M. Sprung, I. A. Vartanyants, Y. S. Meng, O. G. Shpyrko

**Affiliations:** a Department of Physics, University of California San Diego, La Jolla, CA 92093-0319, USA; b Deutsches Elektronen-Synchrotron DESY, Notkestrasse 85, 22607 Hamburg, Germany; c Department of NanoEngineering, University of California San Diego, La Jolla, CA 92093-0448, USA; RIKEN SPring-8 Center, Japan

**Keywords:** extra-thick battery cathodes, Bragg coherent X-ray diffractive imaging, battery cathodes, Bragg diffraction, sphere of confusion, 3D mapping

## Abstract

In Bragg coherent diffractive imaging, the precise location of the measured crystals in the interior of the sample is usually missing. Obtaining this information would help the study of the spatially dependent behavior of particles in the bulk of inhomogeneous samples, such as extra-thick battery cathodes. This work presents an approach to determine the 3D position of particles by precisely aligning them at the instrument axis of rotation. In the test experiment reported here, with a 60 µm-thick LiNi_0.5_Mn_1.5_O_4_ battery cathode, the particles were located with a precision of 20 µm in the out-of-plane direction, and the in-plane coordinates were determined with a precision of 1 µm.

## Abstract 2

A method to determine the 3D position of particles in Bragg coherent diffractive imaging experiments is proposed. Test measurements demonstrate depth-resolution with a precision of 20 µm along the beam.

## 1. Introduction

Imaging of local strain in the bulk of polycrystalline samples requires a probe with high penetration depth and sensitivity to the crystal structure deformations at the nanoscopic level. This became possible with major advances in synchrotron instrumentation, in particular coherent scattering methods that have been developed within the last two decades. Bragg coherent diffractive imaging (CDI) (Robinson *et al.*, 2001 ▸; Miao *et al.*, 2002 ▸; Pfeifer *et al.*, 2006 ▸; Robinson & Harder, 2009 ▸) is now established as a powerful tool for imaging the structure deformation and structure defects in individual nanocrystals (Ulvestad *et al.*, 2015 ▸; Kim *et al.*, 2021 ▸). As the crystals are usually diverse, several particles at different locations are measured to collect sufficient statistical information across the sample (Singer *et al.*, 2018 ▸). The precise location of the measured particles is usually unknown in such experiments, and thus homogeneity of the sample is often assumed.

For systems in which the material response is not uniform, it is important to obtain the location information. For example, in Li-ion batteries with extra-thick cathodes, the charging behavior is expected to depend on the depth under the cathode surface (Zheng *et al.*, 2012 ▸; Lee *et al.*, 2018 ▸). Strengthening the capabilities of operando Bragg CDI with the possibility to map the measured particles would provide a missing link between the performance of individual nanoparticles and the 3D structure of extra-thick electrodes. As a general solution to this problem, here we suggest a method to determine the 3D position of the measured particles in Bragg CDI experiments.

Our method has some similarities with the procedure involving the detection of rotational centers from cross-correlations in micro-tomography (Pan *et al.*, 2012 ▸), which uses optical contrast as a measure of alignment, whereas our work uses the integrated intensity of a Bragg diffraction spot. The fundamental difference is that in tomography the full 180° angular range is available for sample rotations. In Bragg CDI, each particle is observed only in the angular range of about 1° in which the Bragg condition is satisfied. Despite this limitation on the angular range, in which the particle is observed in the beam, our method allows us to determine where exactly along the beam the particle is. This adds new information to the Bragg CDI method.

## 2. Method

**Figure 1:**
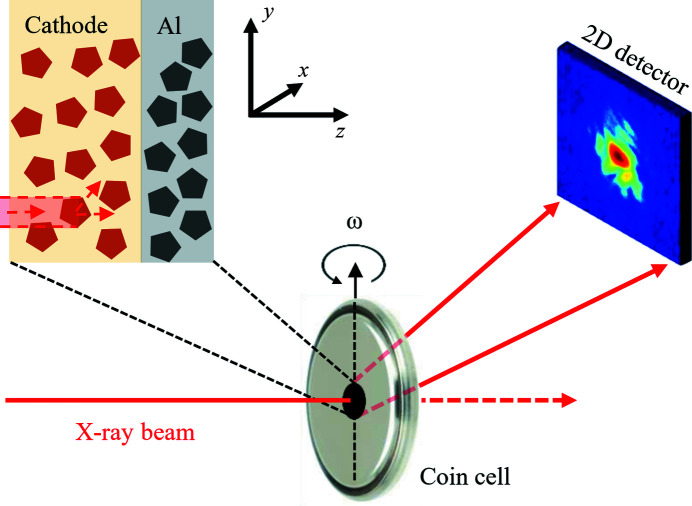
Schematic of the depth-resolved Bragg CDI approach. Before the measurements, the axis of rotation of the instrument (ω) is aligned to intersect with the focused X-ray beam. The sample (in our case, a battery cathode in a coin cell) is searched for particles that have bright and isolated Bragg peaks on the 2D detector. We use the right-hand coordinate system with the *z* axis along the direction of the X-ray beam and the *y* axis aligned with the sample rotation axis.

In a typical Bragg CDI experiment, a finite crystalline sample is illuminated with a focused X-ray beam and oriented to fulfill the Bragg diffraction condition (Vartanyants & Yefanov, 2015 ▸). The resulting far-field interference pattern around the Bragg peak is recorded with a 2D detector (see the schematic in Fig. 1 ▸). Different cross-sections through reciprocal space are accessed by rocking the sample in the beam so that a typical dataset consists of a few tens of diffraction patterns (Williams *et al.*, 2003 ▸). In such a geometry, the crystal is on the instrument axis of rotation aligned to be perpendicular to the X-ray beam. Since the particle must stay in the beam during the scan, bringing the particle precisely to the instrument axis of rotation is an important step.

**Figure 2:**
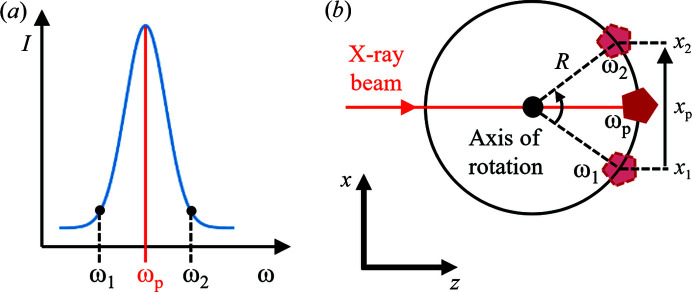
Alignment for the longitudinal position in the depth-resolved Bragg CDI. (*a*) After the *x* and *y* coordinates have been aligned, the Bragg peak intensity is scanned with the ω-scan rotation providing the rocking curve data. Then, *x* profiles are measured on the opposite sides of that curve: ω_1_ and ω_2_. (*b*) If the particle was seated off the axis of rotation, the *x* position of the peak would shift between these two scans. The misalignment along the beam direction is calculated as the amount of that shift divided by the angular difference expressed in radians.

To describe the alignment procedure, let us assume that the nanoparticle is initially located off the axis of rotation. First, we adjust the rotation of the sample (ω) and the translations perpendicular to the beam (*x* and *y*) to maximize the diffracted intensity of the peak. Then, ω is scanned near the position of the highest intensity (ω_*p*_), as shown in Fig. 2 ▸(*a*). For two angular positions on the opposite sides of the resulting intensity profile (ω_1_ and ω_2_), we maximize the intensity by scanning the *x* spatial position. By that we determine how much the sample moved [see Fig. 2 ▸(*b*)]. Finally, we move the sample along the beam by 

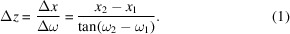

These steps are repeated until Δ*z* = 0, which means the particle is located exactly at the axis of rotation.

## 3. Experiment

We performed our experiment at the P10 Coherence Applications beamline of PETRA III facility (Hamburg, Germany), using an X-ray beam with a photon energy of 11.89 keV that was focused to a 2 µm × 2 µm spot. The sample was an Li-ion battery cathode consisting of a roughly 60 µm-thick layer of LiNi_0.5_Mn_1.5_O_4_ (LNMO) material deposited on a roughly 20 µm-thick Al foil. The cathode was mounted in a way that the X-ray beam was incident normal to the front side (LNMO surface) leaving Al foil downstream as shown in Fig. 1 ▸.

**Figure 3:**
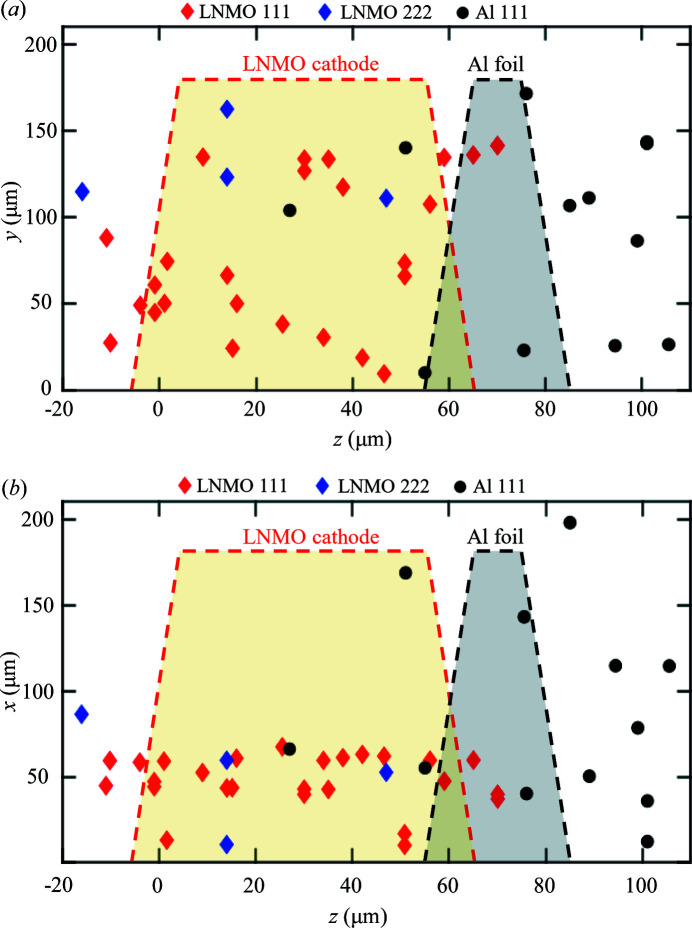
Measured 3D positions of particles in a 60 µm-thick battery cathode. The cathode consisted of the LNMO material (yellow area), deposited on 20 µm-thick Al foil (gray area). Slopes of the red and black dashed contours illustrate the thickness uncertainties for the LNMO and Al layers, respectively, which are estimated to be about 10 µm on each side. LNMO (111) (red markers) and LNMO (222) (blue markers) reflections were used. For Al, we used the (111) reflection (shown by black markers). (*a*) *yz* projection. (*b*) *xz* projection.

First, we selected an Al (111) reflection and measured 12 particles for positional reference to use our mapping method. After that, we switched to the LNMO (111) reflection and measured 30 LNMO particles. Then, to confirm that choice of reflection does not affect the consistency of the results, we switched to the LNMO (222) reflection and measured four more particles. The resulting 3D positions are presented in Figs. 3 ▸(*a*) and 3 ▸(*b*). We can see that the *z* positions of LNMO particles fit well into the 60 ± 20 µm range. The Al particles appear within the expected size of the sample and fit into the 20 ± 30 µm range. The positions of Al particles are less precise due to the much lower quality of the Bragg peaks that were less intense and would often overlap. This can be explained by the significantly smaller grain size of Al. Overall, the positions are consistent with the cathode geometry and dimensions.

We did not apply any bias when choosing the particles and selected them using only two criteria. The first was that there should be no peak overlap with other particles in the beam, and this should not depend on the position in the sample because the sample material was pristine and therefore uniform. The second was high peak intensity, which should also not depend on the position, as the total optical path would be roughly the same for any position along the beam, at least for scattering angles in the range 10–15°, used in our work. Using equation (1), the precision of the positioning in our method can be estimated from the angular difference Δω (0.8° in our case) and the precision of the measurement of Δ*x* (about 0.3 µm in our case). This provides 20 µm precision along the beam, which is confirmed by the experimental data presented in Figs. 3 ▸(*a*) and 3 ▸(*b*).

Instrumental inaccuracies of the rotational and translational positioning can potentially bring systematic errors to our method. At the P10 beamline, we used a six-circle Huber diffractometer with high-precision translational piezo mechanisms. Beforehand, for the measurements, we aligned the beam and the axis of rotation using a metal pin with sharp edges. Considering that all sample rotations in the method were performed within a narrow range of about 1°, we can confidently state that, throughout the measurements, the axis of rotation remained in the beam center with a precision better than 1 µm. Therefore, instrumental errors did not contribute significantly in our case.

## 4. Discussion and conclusions

In our experiment, it was difficult to find particles with isolated and intense Bragg peaks because the diffraction rings from such a thick sample were densely populated with peaks. In future work, this can be mitigated by employing a smaller X-ray beam and choosing higher-order reflections. We note that the low transmission of X-rays in thick samples makes alignment difficult, thus limiting the applications of our method. Future advances in coherent scattering methods toward using higher photon energies would help overcome this problem.

We suggest that our method can be used to explore the difference in charging behaviors of underlying nanoparticles at different depths in extra-thick Li-ion battery cathodes. On one hand, such cathodes are significantly thicker than the depth-resolution of the method (20 µm). On the other hand, they are still thin enough for a hard X-ray beam to penetrate. Such an experiment could help explain the origin of thick electrode electrochemical performance deterioration.

We proposed a simple method for mapping the 3D position of particles in Bragg CDI experiments, which is based on the precise alignment of the selected particles with the instrument axis of rotation. We carried out an experiment in which positions of LiNi_0.5_Mn_1.5_O_4_ battery cathode particles were measured with a precision of 1 µm in the plane perpendicular to the beam and 20 µm in the direction along the beam. In future experiments, our method can help to study inhomogeneous systems in which the structural behavior of composing nanocrystals depends on their location in the sample interior.

## References

[bb1] Kim, Y. Y., Keller, T. F., Goncalves, T. J., Abuin, M., Runge, H., Gelisio, L., Carnis, J., Vonk, V., Plessow, P. N., Vartaniants, I. A. & Stierle, A. (2021). *Sci. Adv.* **7**, 757. 10.1126/sciadv.abh0757 PMC10938497 34597137

[bb2] Lee, B.-S., Wu, Z., Petrova, V., Xing, X., Lim, H.-D., Liu, H. & Liu, P. (2018). *J. Electrochem. Soc.* **165**, A525–A533.

[bb3] Miao, J., Ishikawa, T., Johnson, B., Anderson, E. H., Lai, B. & Hodgson, K. O. (2002). *Phys. Rev. Lett.* **89**, 088303. 10.1103/PhysRevLett.89.088303 12190506

[bb4] Pan, Y., De Carlo, F. & Xiao, X. (2012). *Proc. SPIE*, **8313**, 645–653.

[bb5] Pfeifer, M. A., Williams, G. J., Vartanyants, I. A., Harder, R. & Robinson, I. K. (2006). *Nature*, **442**, 63–66. 10.1038/nature04867 16823449

[bb6] Robinson, I. & Harder, R. (2009). *Nat. Mater.* **8**, 291–298. 10.1038/nmat2400 19308088

[bb7] Robinson, I. K., Vartanyants, I. A., Williams, G., Pfeifer, M. & Pitney, J. (2001). *Phys. Rev. Lett.* **87**, 195505. 10.1103/PhysRevLett.87.195505 11690423

[bb8] Singer, A., Zhang, M., Hy, S., Cela, D., Fang, C., Wynn, T., Qiu, B., Xia, Y., Liu, Z., Ulvestad, A., Hua, N., Wingert, J., Liu, H., Sprung, M., Zozulya, A. V., Maxey, E., Harder, R., Meng, Y. S. & Shpyrko, O. G. (2018). *Nat. Energy*, **3**, 641–647.

[bb9] Ulvestad, A., Singer, A., Clark, J. N., Cho, H. M., Kim, J. W., Harder, R., Maser, J., Meng, Y. S. & Shpyrko, O. G. (2015). *Science*, **348**, 1344–1347. 10.1126/science.aaa1313 26089511

[bb10] Vartanyants, I. A. & Yefanov, O. M. (2015). *X-ray Diffraction. Modern Experimental Techniques*, ch. 12, pp. 341–384. Singapore: Pan Stanford Publishing.

[bb11] Williams, G. J., Pfeifer, M. A., Vartanyants, I. A. & Robinson, I. K. (2003). *Phys. Rev. Lett.* **90**, 175501. 10.1103/PhysRevLett.90.175501 12786079

[bb12] Zheng, H., Li, J., Song, X., Liu, G. & Battaglia, V. S. (2012). *Electrochim. Acta*, **71**, 258–265.

